# Research in the Field of Exercise and Metabolomics: A Bibliometric and Visual Analysis

**DOI:** 10.3390/metabo12060542

**Published:** 2022-06-14

**Authors:** Zhen Lv, Zhi-Gang Gong, Yong-Jiang Xu

**Affiliations:** 1 Key Laboratory of Aquatic Sports Training Monitoring and Intervention of General Administration of Sport of China, Faculty of Physical Education, Jiangxi Normal University, No. 99 Ziyang Avenue, Nanchang 330022, China; 15954811987@163.com; 2 School of Food Science and Technology, Jiangnan University, 1800 Lihu Road, Wuxi 214122, China; yjxutju@gmail.com

**Keywords:** metabolomics, exercise, research frontier, bibliometric analysis, Web of Science

## Abstract

The aim of this article was to conduct a bibliometric analysis of global research trends in the field of exercise and metabolomics between 2005 and 2020. Systematic articles were obtained from the literature in the Web of Science core collection database from 2005 to 2020. The relationship between the number of publications, citations, countries, journals, authors, and the evolution of research hotspots was analyzed. A total of 807 studies were included in the analysis. From 2005 to 2020, the number of citations and the number of published articles showed an upward trend. Keyword co-occurrence indicates that research hotspots are focused on exercise, physical activity, metabolomics, obesity, insulin resistance, inflammation, and cardiovascular disease. Keyword clustering indicates that the research frontier is focused on the field of sports medicine, which includes molecular-level studies of exercise interventions in disease and studies of the physiological mechanisms by which exercise alters the body. Overall, this trinity of models, combining chronic disease with exercise interventions and molecular-level studies of metabolomics, has become the forefront of research in the field. This historical review of the field of exercise and metabolomics will further provide a useful basis for hot issues and future development trends.

## 1. Introduction

In 1999, Professor Nicholson in England first proposed the concept of metabolomics, which defined metabolomics as “the quantitative measurement of the dynamic multi-parametric metabolic response of living systems to pathophysiological stimuli or genetic modification” [1]. Tang (2006) proposed a precise definition: metabolomics is a discipline that provides a quantitative understanding of endogenous metabolites in organisms and the dynamic responses under the influence of changes in endogenous and exogenous factors [2]. Biological compounds at the metabolite level are the most interesting to study in the field of exercise [3]. The use of metabolomics allows for the simultaneous monitoring of hundreds of metabolites, which is particularly valuable when focusing on complex interactions during exercise or nutritional interventions [4], and metabolomics is increasingly being used in sports nutrition research [5]. Current research in the field of exercise and metabolomics is mainly concerned with competitive sports training, sports nutrition, the effects of acute and chronic exercise on body metabolism, the influence of exercise on chronic diseases, and doping [6]. Metabolomics has been applied to various aspects related to exercise, but there is no clear literature indicating research in the field of exercise and metabolomics, and there is a lack of systematic analysis of the field of exercise and metabolomics. Therefore, it is important to summarize previous research progress in the field and use objective data to find the field’s frontiers and lay the foundations for future research.

Research frontiers include hotspots, priorities, and innovations in certain fields [7]. We can discover research frontiers based on bibliometric methods. Research frontiers include explicit frontiers and implicit frontiers. Explicit frontiers are more likely to be taken seriously. We can generally find hotspots and important points in the research field from explicit frontiers [8]. Implicit frontiers, however, are those that are not taken seriously by researchers and may attract attention after a number of years or remain obscure. Implicit frontiers are less visible than explicit frontiers, but their discovery is most valuable [9]. Therefore, frontiers in research should be explored in both explicit and implicit ways.

Bibliometrics is a quantitative method, used to conduct research on qualitative characteristics [10]. Wang et al. published a bibliometric analysis of exercise for lower back pain from 1980 to 2018 [11]. At present, there is no bibliometric analysis of the field of exercise and metabolomics. Therefore, the aim of this study was to summarize the research progress and trends in the field of exercise and metabolomics from 2005 to 2020 through a bibliometric analysis.

## 2. Results

A total of 807 articles were retrieved, with a total of 16,730 citations, 15,408 citations excluding self-citations, an average of 20.73 citations per item, and an H index of 59.

### 2.1. Analysis of Number of Citations, Number of Publications

**Figure 1:**
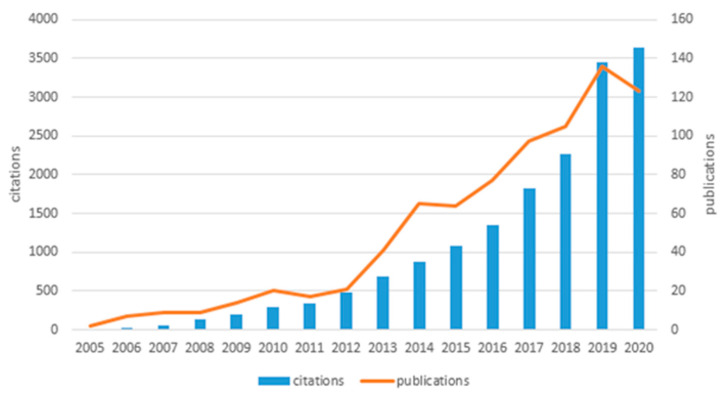
Number of citations per year and number of papers published per day.

The number of papers cited and the number of papers published (Figure 1) showed a relatively stable upward trend during from 2006 to 2018; the number of papers published during from 2012 to 2014 showed an especially rapid upward trend, indicating that researchers’ attention to the field of exercise and metabolomics has increased year by year. The number of citations and publications showed a sharp upward trend during the period 2018–2019, indicating that the field has gradually become a research hotspot. The number of citations decreased during 2019–2020 compared to 2018–2019. The number of publications in 2020 was less than the number of papers published in 2019. Both of these correlate with the cut-off time for the data search.

### 2.2. Analysis of Periodicals

Table 1 was generated from the Web of Science self-contained data using Microsoft Excel 2016. Of the 807 articles included in the analysis, the top three journals all had more than 30 publications, with 5.081% of the 807 articles in METABOLOMICS in first place. In terms of journal affiliation, 50% of the top 10 journals were from the USA, 20% from Switzerland and Germany, respectively, and 10% from the UK. It is evident that the relevant journals in the USA are very focused on this field and that METABOLOMICS has a high focus on research in the field of exercise and metabolomics.

### 2.3. Analysis of Country/Region

A total of 807 papers were published, from 59 countries/regions. The United States, China, England, and Germany are the countries with the highest level of research in the field of exercise and metabolomics (Table 2). The number of publications over 100 is concentrated in the USA, China, and England. The reasons for this may be related to the conference entitled “Metabolic Profiling: Pathways in Discovery” held in the USA in 2001, the support of the Dalian Institute of Chemical Physics of the Chinese Academy of Sciences for the direction of metabolomics during the disciplinary planning in 2001, and the Fourth Conference on Plant Metabolomics held in the UK in 2006 [12]. These conferences have accelerated the development of metabolomics and laid a solid foundation for research in the field of metabolomics and exercise.

### 2.4. Analysis of Research Direction

There are 66 research directions in the 807 publications, of which the biochemistry–molecular biology direction is the first among, with 180 publications, followed by chemistry and endocrine metabolism. The high productivity of the literature is concentrated in molecular-related fields, which are closely related to the research object (metabolome).

### 2.5. Analysis of Author

**Figure 2:**
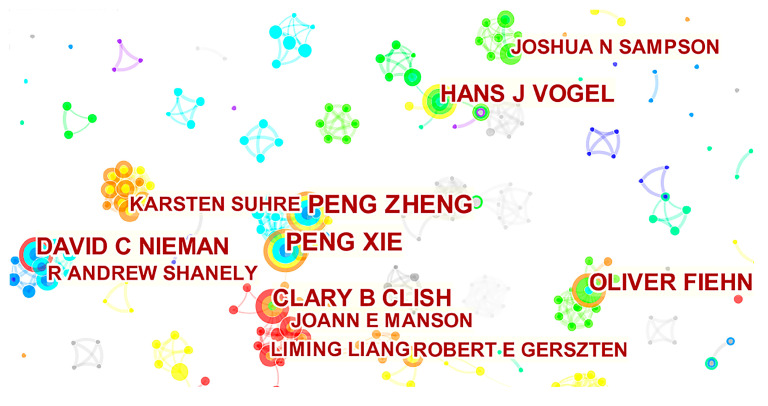
Visualization map of highly cited authors in the field of exercise and metabolomics (top 10 cited authors).

**Figure 3:**
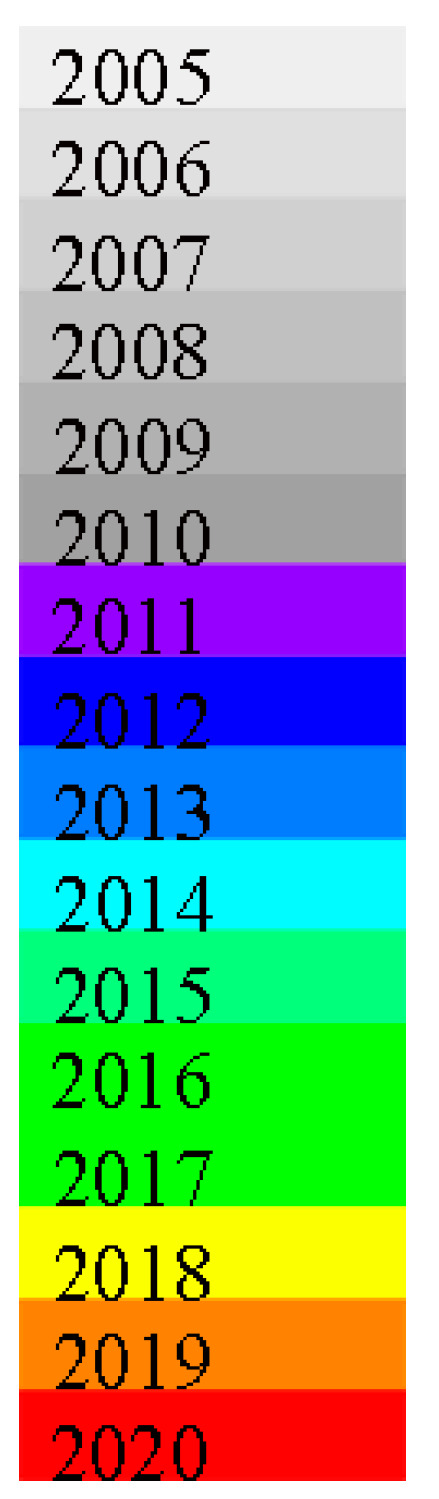
Color–time correspondence legend.

The authors of the 807 publications were analyzed in two ways, examining both the highly productive authors and the highly cited authors in the field. This not only provides an insight into the international authors that are active in fields related to exercise and metabolomics but also allows for the identification of authors with an influence in the field. The graph of high-yielding authors (≥5 publications) in the field of exercise and metabolomics (Figure 2. See Tables S2 and S3 for details.) was obtained using CiteSpace analysis. Larger nodes in Figure 2 indicate authors with more publications, where the connecting lines between nodes indicate collaborations and their colors indicate the time of the first collaboration. The color of the node chronology gradually changes from white to red, corresponding to the time of appearance from top to bottom (Figure 3). High producers in this area (Figure 2) are PENG ZHENG (10), PENG XIE (10), HANS J VOGEL (8), CLARY B CLISH (8), DAVID C NIEMAN (8), OLIVER FIEHN (8), LIMING LIANG (5), JOSHUA N SAMPSON (5), ROBERT E GERSZTEN (5), KARSTEN SUHRE (5), R ANDREW SHANELY (5) and JOANN E MANSON (5). The team of PENG ZHENG and PENG XIE published six papers between 2013 and 2014 and four papers between 2018 and 2019. Although the team has not published any core literature in the field of exercise and metabolomics for 3 years, these data can also indicate a high level of interest in this area of research. The team with CLARY B CLISH at its core published 2, 3, and 3 papers each in 2017, 2019, and 2020, indicating that the team is at the forefront of research in this area.

**Figure 4:**
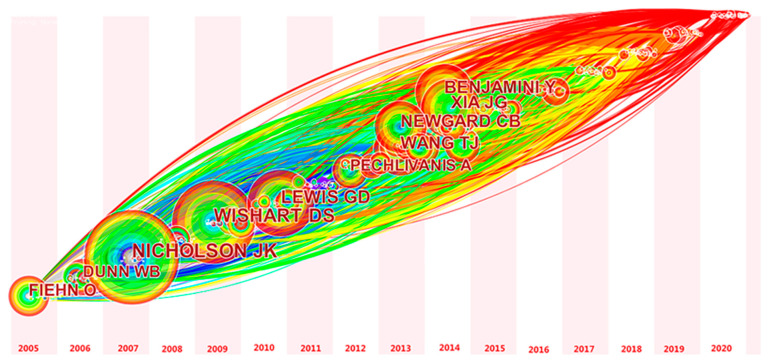
Time zone diagram of co-cited analysis of authors in the field of exercise and metabolomics (top 10 cited frequencies).

The time zone map of author co-citation analysis (top 10 citation frequency) (Figure 4) was obtained using CiteSpace analysis. On this time zone graph spanning 2005–2020, each time zone represents a year, where authors with the same first citation time are placed in the same time zone. The graph shows the trend in time to the first citation of cited authors, from the bottom left to the top right. The larger the node in Figure 4, the more frequently the author is cited. The top 10 cited authors (Figure 4) are Nicholson JK (115), Wishart DS (100), Lewis GD (81), Xia JG (71), Benjamini Y (69), Wang TJ (64), Newgard CB (62), Fiehn O (51), Dunn WB (48), and Pechlivanis A (44). Foreign scholars have a greater influence in the field of exercise and metabolomics research compared to domestic scholars.

From a comprehensive perspective, the three domestic scholars, PENG ZHENG, PENG XIE, and LIMING LIANG, have published more articles in the field of exercise and metabolomics research, indicating their increased attention to this field. The top 10 citation frequencies are all foreign scholars, indicating that foreign scholars have a greater influence on research in this field.

### 2.6. Analysis of Explicit Frontier

**Figure 5:**
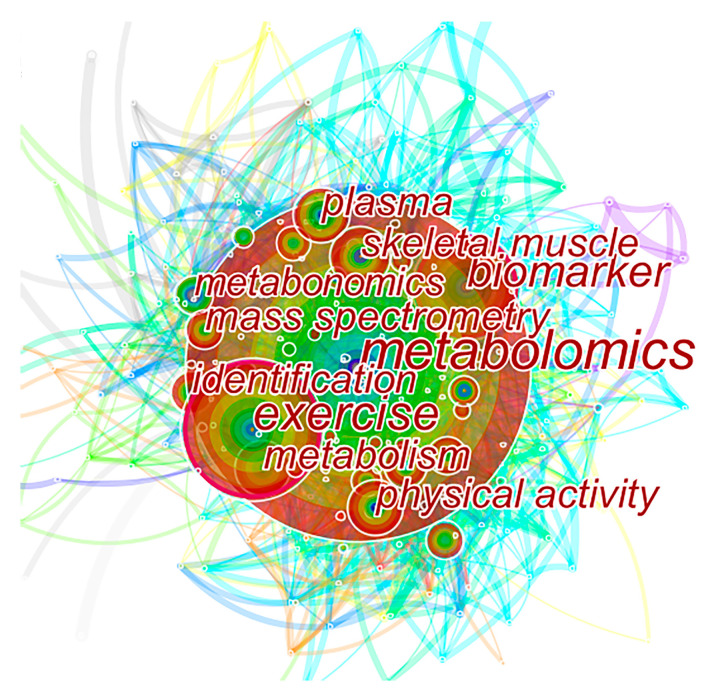
Visualization map of word-frequency distribution of keywords in the field of exercise and metabolomics (top 10 frequency).

A visualization of the keyword word-frequency distribution of studies in the field of exercise and metabolomics was formed through CiteSpace (Figure 5) and a comprehensive table of keyword word-frequency and centrality distribution of studies in the field of exercise and metabolomics (Table 3). No similar keywords were combined in the formation process. The graphs and tables were formed to uncover the general characteristics and research frontiers in the field of exercise and metabolomics.

In the keyword co-occurrence visualization mapping construction, the time was set from 2005 to 2020, the time slice was 1 year, and the Top N (*N* = 50) method was chosen. A module value (Q) > 0.3 in CiteSpace was considered to be significant in terms of clustering structure. The clustering was considered reasonable when the average profile value (S) was >0.5, and plausible when S was >0.7 [13]. In this paper, the Q and S values are 0.3283 and 0.7227, respectively, indicating that the constructed keyword clustering map is reasonable and has a high degree of confidence.

The node size in the keyword co-occurrence graph reflects the frequency of keywords, and its size is proportional to keyword frequency. The smaller the node, the less frequently the keyword appears and the less attention it receives [14].

Figure 5 and Table 3 show that the main research hotspots in the field of exercise and metabolomics include exercise, physical activity, metabolomics, obesity, insulin resistance, inflammation, and cardiovascular disease. Studies have mostly used mass spectrometry and metabolic profiling methods. Plasma, serum, skeletal muscle, and urine samples are analyzed to identify biomarkers of amino acids and metabolites that are affected by exercise interventions. The identified biomarkers are useful not only for determining the risk of disease in advance, but also for the diagnosis of the disease.

### 2.7. Analysis of Implicit Frontier

**Figure 6:**
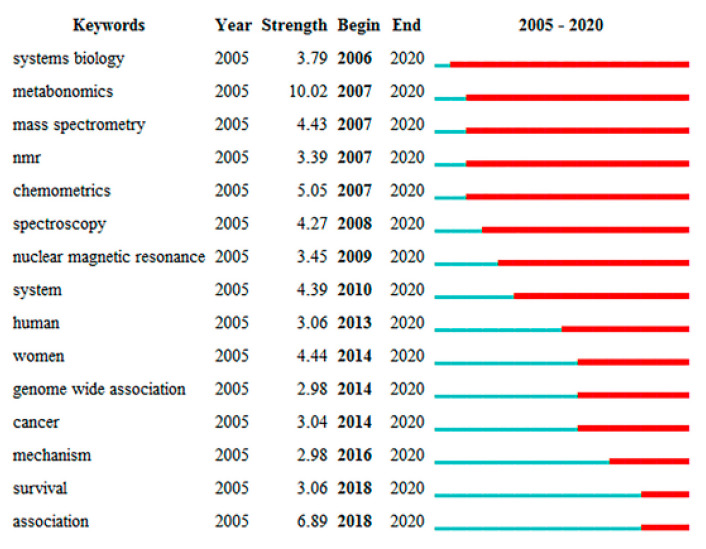
Visualization map of highly burst terms in the filed of exercise and metabolomics (top 15 burst intensity). The blue line indicates the timeline, and the red segment on it indicates the duration of the burst.

The visualization of the highly burst term in the field of exercise and metabolomics was generated by CiteSpace (Figure 6). The top 15 keywords with the highest citation burst emerged after 2005, all with different start dates, but the emergence did not end until 2020, suggesting that these keywords continued to receive attention in the field of exercise and metabolomics. Systems biology was the first to start appearing and has been appearing for the longest time, suggesting that this keyword was noticed early and has received long-term attention. Metabonomics has the highest burst strength, followed by association. Association has emerged more recently but exceeds the strength of the other 13 keywords. This may be related to the analysis of the association between disease and metabonomics through the study of metabonomics in certain chronic diseases in recent years, which correspond to the conclusions obtained in the previous analysis of high-frequency keywords. In conclusion, this trinity of models combining chronic disease with exercise interventions and molecular-level studies of metabolomics has become the forefront of research in the field.

### 2.8. Analysis of Dynamic Development Trend

**Figure 7:**
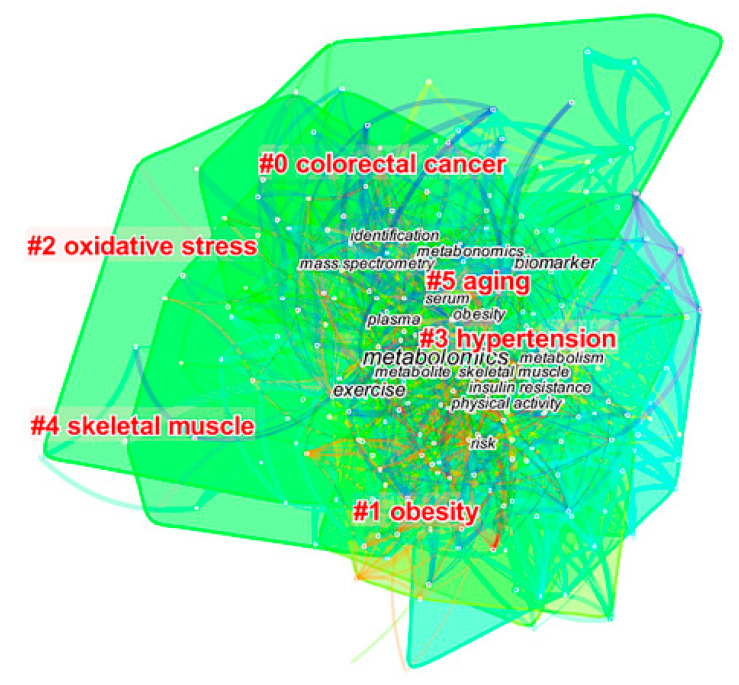
Visualization map of keyword clustering in the field of exercise and metabolomics.

The ordering of the clusters is determined by the number of nodes contained within them; the higher the number of nodes, the smaller the cluster number. From #0 to #5, a total of 5 clusters were generated (Figure 7). The colors of the different clusters indicate the different times at which the clusters emerged, and the specific year of cluster emergence can be determined by the year color axis (Figure 3). Detailed information is provided for the keyword clusters, such as the number of nodes, profile values, year of formation, tag words, and the main high-frequency keywords within the clusters (Table 4). The clustering of the years after 2010 may be related to the participation of Professor Nicholson, the founder of metabolomics, in the 2010 Nature article ‘2020 Visions’, which provided a major boost to the development of metabolomics and deepened the integration of exercise and metabolomics.

The field of exercise and metabolomics research is focused on two hot research clusters (Figure 7 and Table 4. See Table S1 for details), divided by the object of study, including studies of disease and studies of the physiological mechanisms of the organism. Study cluster 1 focuses on chronic diseases, including colorectal cancer, obesity, and hypertension. Study cluster 2 focuses on physiological mechanisms, including oxidative stress, skeletal muscle, and aging. Study cluster 1 focuses on the molecular level of exercise interventions in disease, while study cluster 2 focuses on the physiological mechanisms by which exercise alters the body. Both study clusters use metabolomics as a bridge to explore the relevant components of metabolites, gain insight into micro-level influences and provide empirical evidence for macro-level changes.

## 3. Discussion

The combination of the fields of metabolomics and exercise has assisted scholars involved in deeper research. The current analysis was based on 15 years of published literature in the field of exercise and metabolomics. A total of 807 publications were obtained according to the search strategy, and the bibliometric indicators (number of publications, number of cited articles, authors, keywords, and other items) of the search results were visualized and analyzed by Microsoft Excel 2016 and CiteSpace software.

The results of this study provide a systematic narrative of the field of exercise and metabolomics, with a major focus on the hotspots and future trends related to the field. The results show that, first, the number of publications and citations showed a steady increase, indicating that the field of exercise and metabolomics is gradually becoming a research hotspot. Secondly, related journals in the United States pay great attention to this field. Third, three countries, the United States, China, and England, have a high scientific research level in this field. Fourth, the research direction of this field is mainly focused on molecular-science-related fields. Fifth, three Chinese scholars, PENG ZHENG, PENG XIE, and LIMING LIANG, have more publications in the field of exercise and metabolomics research, which indicates that they pay more attention to this field. The top 10 citation frequencies are all foreign scholars, which indicates that foreign scholars have a greater influence on the research in this field. Sixth, from the keyword co-occurrence analysis, the research hotspots in this field are mainly focused on exercise, physical activity, metabolomics, obesity, insulin resistance, inflammation, and cardiovascular disease. Seventh, from the keyword burst analysis, the trinity model of chronic diseases combined with exercise interventions, complemented by metabolomics research at the molecular level, has become a research hotspot in the field of exercise and metabolomics. Eighth, from the keyword clustering analysis, the field of exercise and metabolomics research is focused on two hot study clusters, with study cluster 1 focusing on the molecular level of exercise interventions in disease and study cluster 2 focusing on the physiological mechanisms by which exercise alters the body. The examples of study cluster 1 and study cluster 2 are as follows.

Using the example of obesity in research cluster 1: obesity is a growing epidemic in the population and can be harmful to health, for example, leading to an increase in cardiovascular disease, type 2 diabetes, and cancer mortality [15,16,17]. Exercise training is a common treatment known to combat the metabolic consequences of obesity. Studies have shown that metabolomics can effectively demonstrate obesity-induced metabolic disturbances and support the claim that exercise training improves this condition [18]. In studies of exercise intervention in overweight individuals, intrahepatic glycine binding was found to be more effective in eliminating excess acyl from branched-chain and aromatic amino acid metabolism, rather than increasing branched-chain amino acid processing through oxidation and cycling, and this mechanism may play a role in modulating the relationship between exercise, branched-chain amino acid metabolism, and insulin sensitivity [19]. Zheng H (2014) showed that there was no association between adolescent metabolites and physical activity during adolescent development in overweight adolescents aged from 12 to 15 years [20]. The findings of this study provide a reference for the prevention of adolescent obesity. These obesity-related studies are centered around the same research idea. The idea is that researchers use metabolomics as a tool and exercise as an intervention to explore the metabolites produced in the process of chronic disease to the point where the intervention has an effect in more depth, thus discovering the mechanisms of chronic disease at the molecular level and the biomarkers that can be found in the process, which provides a reliable basis for the prevention and prognosis of the development of obesity.

Using the example of oxidative stress in research cluster 2: at present, oxidative stress is a condition in which a transient or chronic elevation in the concentration of reactive oxygen species occurs under steady-state conditions, thereby disrupting cellular metabolism and its regulation, and ultimately leading to the destruction of cellular components [21]. It is well-known that intense exercise can exacerbate stressors in the body, such as oxidative stress and inflammation, with the latter occurring at both the muscula [22,23,24] and gastrointestinal (GI) [25,26,27] levels. However, Zannoni A (2020) [28] used metabolomics to assess potential stress markers in fecal samples from dogs after intense exercise and found that exercise was a mild stressor stimulus in the model that he set up. Chorell E (2012) [29] found, from the process of oxidative stress biomarker changes, that participants in a state of high physical fitness showed increased cardiopulmonary inflammation and antioxidant defenses, which can more easily handle the inflammatory response that arises after low levels of exercise and excessive energy intake. In contrast, DeBalsi K L (2014) [30] found that the deletion of thioredoxin-interacting protein leads to muscle-specific substrate oxidative damage through targeted metabolomics, which results in decreased exercise endurance. These studies use specific biomarkers as a tool to explore the ideas regarding interactions between exercise and organismal mechanisms at the molecular level. The same research ideas were used by Couto M (2017) [31] and Silva D (2019) [32]. Nieman D C(2012) [5] and Saoi M (2019) [33] investigated the effect of pre-exercise intake of different substances on the molecular level of oxidative stress in the organism, respectively. Al-Khelaifi F (2018) [34] and Nieman D C (2014) [4] contributed to studies related to oxidative stress using metabolomics, with the former identifying oxidative stress-related metabolites in high-strength and high-endurance athletes and the latter identifying biomarkers of oxidative stress through the athletes’ plasma after a 75 km cycling race. These studies suggest that the use of metabolomics techniques can directly explore the alterations in the physiological mechanisms of the athlete’s body as well as exploring the effects of pre- and post-exercise substance intake on the molecular level of body mechanisms, which provides a basis for research ideas for future studies. In summary, these studies explored the relationship between both exercise and body mechanisms using a metabolomic approach and explored the effects of alterations in one on the other, thus investigating the mechanisms of the interaction between exercise and body mechanisms.

The emergence of metabolomics methods has provided new approaches to the study of chronic diseases and physiological mechanisms of the organism in the field of exercise. These research methods have a great reference value for relevant researchers. Therefore, research in the field of exercise and metabolomics has broad prospects.

### Strengths and Limitations

This study is the first bibliometric analysis based on the WOS database summarizing worldwide research hotspots and trends in the field of exercise and metabolomics over the last 15 years. In addition to analyzing the number of publications, cited papers, journals, countries, and authors, this study also analyzes the co-occurrence, burst, and clustering of keywords in the field in terms of dominant and implicit frontiers. This study also has some limitations.

(1) The search strategy was limited to the Web of Science Core Database and only articles were searched. Therefore, these factors may lead to publication bias.
(2) Co-citation analysis, as well as the geospatial visualization in CiteSpace, were not used but had no effect on the final analysis results.
(3) The search time was not comprehensive enough, resulting in four articles from the Web of Science Core Database before 2005 not being included in the analysis, but this did not affect the final analysis results.

## 4. Materials and Methods

### 4.1. Search Strategy

The data in this paper are based on the Web of Science Core Collection, including SCI-EXPANDED (2001–2020), SSCI (2001–2020), A&HCI (2003–2020), ESCI (2015–2020), CCRE-EXPANDED (1985–2020) and IC (1993–2020). The database is searched with “metabolome” OR “metabonomics” OR “metabolomics” as the subject word, and the search results are refined with “exercise” or “sport” or “physical activity” or “training”. The selected literature type was ARTICLE. There were no restrictions on languages and countries/regions, and the timespan ranged from 2005 to 2020. Data retrieval and download ended on 5 December 2020, and the latest update date in the database was 4 December 2020.

### 4.2. Analysis Tools

Microsoft Excel 2016 was used to tabulate the results (high-yielding countries, journals) from the Web of Science Core Database. This paper used CiteSpace5.7.R2 to draw the knowledge map, and analyzed the map by combining qualitative and quantitative methods to study the hotspots in the field of exercise and metabolomics and analyze their development trends. CiteSpace software uses the lexical analysis method to identify and analyze hotspots and frontiers in the research field. Keywords in the literature are highly distilled and general statements regard the topic and core terms of an article. Keywords are extremely helpful in identifying research hotspots in the scientific field [35]. The detection of high-frequency keywords and burst terms in CiteSpace software helps in the discovery of explicit and implicit frontiers [36]. The keyword clustering method in CiteSpace is based on the principle of co-word analysis, which is essentially “a two-by-two count of the number of occurrences of a group of words in the same document, which is used as a basis for cluster analysis to reflect the relationship between these words” [37]. The co-word analysis method can dig deeper into the dynamic trends in the discipline [38]. Therefore, the use of word-frequency analysis to analyze the explicit and implicit frontiers in the field of exercise and metabolomics research is complemented by co-word analysis to provide insight into the frontiers and development trends in the field.

### 4.3. Data Extraction

After designing the search strategy, the two authors extracted literature and bibliometric information separately. One downloaded the published studies and the other extracted data from the literature (number of publications, number of cited articles, journals, countries, research directions, authors, trends in keywords) using CiteSpace 5.7.R2 and Microsoft Excel 2016. Relevant figures and tables were obtained and data were interpreted by the above-mentioned analysis tools.

The retrieved papers were selected using the Other file format option in Export, and all records (author, title, source publication, and abstract) and references cited in the bibliographic information of the literature were downloaded and saved as plain text files. CiteSpace used these files to generate knowledge maps.

## 5. Conclusions

This analysis of published research in the field of exercise and metabolomics over the last 15 years may prove to be the basis for developing and improving research ideas in the field and further research in sports medicine. This analysis may enable novices in the field to quickly understand and engage in research and may guide relevant research teams. At the same time, this study may promote the collaboration of research teams in the field to explore exercise for health. With the increasing interest in the field of exercise and metabolomics, hot research clusters are increasingly focused on the study of exercise interventions in chronic diseases and metabolism-related molecular changes in the physiological mechanisms by which exercise alters the body. A possible future trend in this field is to continue with an in-depth exploration of both the metabolomics of exercise interventions in chronic diseases and the metabolomics of exercise alterations in physiological mechanisms of the body. Although this study has several limitations, it provides a historical analysis of research in the field of exercise and metabolomics and provides researchers with information on hotspots and development trends in this field.

## Figures and Tables

**Table 1:** Main source journals in the field of exercise and metabolomics (top 10 papers).

Serial Number	Source Publication Name	Number of Posts Issued	Percentage of Published Articles in Total Retrieved Articles (%)	5 Year Impact Factor	Country
1	Metabolomics	41	5.081	3.625	United States
2	Journal of Proteome Research	35	4.337	3.946	United States
3	Metabolites	33	4.089		Switzerland
4	Plosone	29	3.594	3.227	United States
5	Scientific Reports	23	2.85	4.576	Germany
6	Analytical Chemistry	22	2.726	6.642	United States
7	Analytic and Bioanalytical Chemistry	12	1.487	3.444	Germany
8	Amercan Journal of Clinical Nutrition	9	1.115	7.831	United Kingdom
9	Frontiers in Physiology	9	1.115	3.697	Switzerland
10	Journal of Agricultural and Food Chemistry	8	0.991	4.290	United States

Note: The 5 year impact factor data are from the 2019 edition of Journal Citation Reports.

**Table 2:** Main source countries for research in the field of exercise and metabolomics (top 10 papers).

Country/Region	Number of Posts Issued	Percentage of Total Retrieved Articles (%)
USA	275	34.077
China	154	19.083
England	104	12.887
Germany	88	10.905
Canada	60	7.435
Italy	50	6.196
Spain	50	6.196
Japan	42	5.204
France	38	4.709
Australia	36	4.461

**Table 3:** Distribution of word frequency and centrality of research keywords in the field of exercise and metabolomics.

Serial Number	High-Frequency Keywords	Frequency	Serial Number	Highly Central Keywords	Centrality Value
1	Metabolomics	430	1	Exercise	0.16
2	Exercise	197	2	Biomarker	0.11
3	Biomarker	148	3	Metabolism	0.09
4	Metabolism	86	4	Acid	0.07
5	Skeletal muscle	86	5	Profile	0.07
6	Plasma	83	6	Metabolomics	0.06
7	Physical activity	78	7	Skeletal Muscle	0.06
8	Mass spectrometry	76	8	Physical activity	0.06
9	Identification	74	9	Mass spectrometry	0.06
10	Metabonomics	68	10	Amino acid	0.06
11	Obesity	66	11	Urine	0.06
12	Insulin resistance	64	12	Inflammation	0.06
13	Risk	60	13	Cardiovascular disease	0.06
14	Metabolite	56	14	Response	0.06
15	Serum	55	15	Disease	0.05

**Table 4:** Cluster table of research keywords in the field of exercise and metabolomics.

Clustering	Number of Nodes	Contour Value	Year of Formation	Label Words	Keywords in Cluster
0	55	0.761	2012	colorectal cancer	metabolomics, biomarkers, plasma, mass spectrometry, identification, metabonomics, serum, acid, profile, expression, urine, nuclear magnetic resonance, cancer, spectroscopy, chemometrics, system
1	53	0.745	2014	obesity	physical activity, obesity, insulin resistance, risk, disease, amino acid, diet, health, inflammation, association, fatty acid, cardiovascular disease, risk factor, mechanism, human, woman, genome wide association
2	36	0.649	2013	oxidative stress	performance, oxidative stress, muscle, metabolome, model, response, physical exercise, systems biology, stress, liver, mice, brain
3	33	0.677	2014	hypertension	protein, supplementation, NMR spectroscopy, mortality, nutrition, pathway, survival, body mass index, proteomics, magnetic resonance spectroscopy
4	33	0.793	2011	skeletal muscle	exercise, metabolism, skeletal muscle, glucose, validation, gene expression, database, capacity, oxidation, gene, lactate
5	28	0.670	2015	aging	metabolite, rat, blood, prediction, age, gut microbiota, aging, carnitine, lipidomics, targeted metabolomics, Phosphatidylcholine, acylcarnitine

## Supplementary Materials

The following are available online at https://www.mdpi.com/article/10.3390/metabo12060542/s1, Table S1: Keywords (top 10) within clusters related to the papers (top 5), Table S2: Top 20 authors distributed by publications in the field of exercise and metabolomics, Table S3: Top 20 authors distributed by citations in the field of exercise and metabolomics.
