# Supplementary material for: Research in the Field of Exercise and Metabolomics: A Bibliometric and Visual Analysis
Source: Metabolites. 2022 Jun 14;12(6):542. doi: 10.3390/metabo12060542 (PMC9230385; doi:10.3390/metabo12060542)
Supplement: Supplementary file 1 [file metabolites-12-00542-s001.zip › Supplementary Table S1.pdf]

Supplementary Table S1: Keywords (top 10) within clusters related to the papers (top 5)

Cluster Labels	Keyword Time-cited	Keyword (top 10)	Title	First Author	Citations (top 5)	DOI
	430	metabolomics	Quantitative Serum Nuclear Magnetic Resonance Metabolomics in Cardiovascular Epidemiology and	Soininen, Pasi	227	10.1111/j.2047-6310.2012.00087.x
			Circulating branched-chain amino acid concentrations are associated with obesity and future insulin resistance in children and adolescents	McCormack, S E	209	10.1111/j.2047-6310.2012.00087.x
			Serum metabolomics reveals gamma-glutamyl dipeptides as biomarkers for discrimination among different forms of liver disease	Soga, Tomoyoshi	145	10.1016/j.jhep.2011.01.031
			A diabetes-predictive amino acid score and future cardiovascular disease	Magnusson, Martin	139	10.1093/eurheartj/ehs424
			Combined Proteomic and Metabolomic Profiling of Serum Reveals Association of the Complement System with Obesity and Identifies Novel Markers of Body Fat Mass Changes	Oberbach, Andreas	135	10.1021/pr2005555
	148	biomarker	Proposed minimum reporting standards for data analysis in metabolomics	Goodacre, Royston	247	10.1007/s11306-007-0081-3
			The dynamic range of the human metabolome revealed by challenges	Krug, Susanne	189	10.1096/fj.11-198093
			Serum metabolomics reveals gamma-glutamyl dipeptides as biomarkers for discrimination among different forms of liver disease	Soga, Tomoyoshi	145	10.1016/j.jhep.2011.01.031
			Potential metabolite markers of schizophrenia	Yang, J	122	10.1038/mp.2011.131
			A Novel Serum Metabolomics-Based Diagnostic Approach to Pancreatic Cancer	Kobayashi, Takashi	108	10.1158/1055-9965.EPI-12-1033
			A Novel Serum Metabolomics-Based Diagnostic Approach for Colorectal	Nishiumi, Shin	154	10.1371/journal.pone.0040459
			Long-term Leisure-time Physical Activity and Serum Metabolome	Kujala, Urho M	109	10.1161/CIRCULATIONAHA.112.105551

#0 colorectal cancer	83	plasma	Metabolomic profiling of dietary-induced insulin resistance in the high fat-fed C57BL/6J mouse	Shearer, J	94	10.1111/j.1463-1326.2007.00837.x
			Metabolomics in Epidemiology: Sources of Variability in Metabolite Measurements and Implications	Sampson, Joshua N	83	10.1158/1055-9965.EPI-12-1109
			Metabolic and hormonal responses to isoenergetic high-intensity interval exercise and continuous moderate-intensity exercise	Peake, Jonathan M	70	10.1152/ajpendo.00276.2014
	76	mass spectrometry	The dynamic range of the human metabolome revealed by challenges	Krug, Susanne	189	10.1096/fj.11-198093
			A Novel Serum Metabolomics-Based Diagnostic Approach for Colorectal	Nishiumi, Shin	154	10.1371/journal.pone.0040459
			Metabolomic NMR Fingerprinting to Identify and Predict Survival of Patients with Metastatic Colorectal Cancer	Bertini, Ivano	113	10.1158/0008-5472.CAN-11-1543
			A Novel Serum Metabolomics-Based Diagnostic Approach to Pancreatic Cancer	Kobayashi, Takashi	108	10.1158/1055-9965.EPI-12-1033
			A multivariate screening strategy for investigating metabolic effects of strenuous physical exercise in human serum	Pohjanen, Elin	85	10.1021/pr070007g
	74	identification	Proposed minimum reporting standards for data analysis in metabolomics	Goodacre, Royston	247	10.1007/s11306-007-0081-3
			A Novel Serum Metabolomics-Based Diagnostic Approach to Pancreatic Cancer	Kobayashi, Takashi	108	10.1158/1055-9965.EPI-12-1033
			Human metabolic correlates of body mass index	Moore, Steven C	87	10.1007/s11306-013-0574-1
			Virtual Quantification of Metabolites by Capillary Electrophoresis-Electrospray Ionization-Mass Spectrometry: Predicting Ionization Efficiency Without Chemical Standards	Chalcraft, Kenneth R	85	10.1021/ac802272u
			Medium Chain Acylcarnitines Dominate the Metabolite Pattern in Humans under Moderate Intensity Exercise and Support Lipid Oxidation	Lehmann, Rainer	81	10.1371/journal.pone.0011519

68	metabonomics	Investigations of the effects of gender, diurnal variation, and age in human urinary metabolomic profiles	Slupsky, Carolyn M	283	10.1021/ac0708588
		Analysis of metabolomic data using support vector machines	Mahadevan, Sankar	216	10.1021/ac800954c
		Identification and Validation of Urinary Metabolite Biomarkers for Major Depressive Disorder	Zheng, Peng	126	10.1074/mcp.M112.021816
		Distinct Urinary Metabolic Profile of Human Colorectal Cancer	Cheng, Yu	124	10.1021/pr201001a
		International NMR-Based Environmental Metabolomics Intercomparison Exercise	Viant, Mark R	112	10.1021/es802198z
55	serum	Metabolomics as a Tool for Discovery of Biomarkers of Autism Spectrum Disorder in the Blood Plasma of Children	West, Paul R	93	10.1371/journal.pone.0112445
		High-throughput H-1 NMR-based metabolic analysis of human serum and urine for large-scale epidemiological studies: validation study	Barton, Richard H	89	10.1093/ije/dym284
		Metabolomic Profiling of Serum from Human Pancreatic Cancer Patients Using H-1 NMR Spectroscopy and Principal Component Analysis	OuYang, Dong	68	10.1007/s12010-011-9240-0
		Metabolomics provide new insights on lung cancer staging and discrimination from chronic obstructive pulmonary Metabolomics provide new insights on lung cancer staging and discrimination from chronic obstructive pulmonary disease	Deja, Stanislaw	59	10.1016/j.jpba.2014.08.020
		Comparing metabolite profiles of habitual diet in serum and urine	Playdon, Mary C	59	10.3945/ajcn.116.135301
		Tissue imaging and serum lipidomic profiling for screening potential biomarkers of thyroid tumors by matrix-assisted laser desorption/ionization-Fourier transform ion cyclotron resonance mass spectrometry	Guo, Shuai	38	10.1007/s00216-014-7846-0

	45	acid	Metabolomics insights into early type 2 diabetes pathogenesis and detection in individuals with normal fasting glucose	Merino, Jordi	31	10.1007/s00125-018-4599-x
			Bananas as an Energy Source during Exercise: A Metabolomics Approach	Nieman, David C	30	10.1371/journal.pone.0037479
			Effects of exposure to water disinfection by-products in a swimming pool: A metabolome-wide association study	van Veldhoven, Karin	27	10.1016/j.envint.2017.11.017
			Response of Gut Microbiota to Metabolite Changes Induced by Endurance Exercise	Zhao, Xia	26	10.3389/fmicb.2018.00765
	41	profile	A diabetes-predictive amino acid score and future cardiovascular disease	Magnusson, Martin	139	10.1093/eurheartj/ehs424
			Long-term Leisure-time Physical Activity and Serum Metabolome	Kujala, Urho M	109	10.1161/CIRCULATIONAHA.112.105551
			Multivariate Modeling and Prediction of Breast Cancer Prognostic Factors Using MR Metabolomics	Giskeodegard, Guro F	100	10.1021/pr9008783
			Integrating Omic Technologies into Aquatic Ecological Risk Assessment and Environmental Monitoring: Hurdles, Achievements, and Future Outlook	Van Aggelen, Graham	99	10.1289/ehp.0900985
			Metabolomics as a Tool for Discovery of Biomarkers of Autism Spectrum Disorder in the Blood Plasma of Children	West, Paul R	93	10.1371/journal.pone.0112445
	78	physical activity	Metabolic Signatures of Adiposity in Young Adults: Mendelian Randomization Analysis and Effects of Weight Change	Wurtz, Peter	135	10.1371/journal.pmed.1001765
			Human metabolic correlates of body mass index	Moore, Steven C	87	10.1007/s11306-013-0574-1
			Clinical and Metabolic Characterization of Lean Caucasian Subjects With Non-alcoholic Fatty Liver	Feldman, Alexandra	73	10.1038/ajg.2016.318
			Metabolite patterns predicting sex and age in participants of the Karlsruhe Metabolomics and Nutrition (KarMeN) study	Rist, Manuela J	54	10.1371/journal.pone.0183228

			Linking diet, physical activity, cardiorespiratory fitness and obesity to serum metabolite networks: findings from a population-based study	Floegel, A	52	10.1038/ijo.2014.39
	66	obesity	Combined Proteomic and Metabolomic Profiling of Serum Reveals Association of the Complement System with Obesity and Identifies Novel Markers of Body Fat Mass Changes	Oberbach, Andreas	135	10.1021/pr2005555
			Metabolic Signatures of Adiposity in Young Adults: Mendelian Randomization Analysis and Effects of Weight Change	Wurtz, Peter	135	10.1371/journal.pmed.1001765
			Long-term Leisure-time Physical Activity and Serum Metabolome	Kujala, Urho M	109	10.1161/CIRCULATIONAHA.112.105551
			Human metabolic correlates of body mass index	Moore, Steven C	87	10.1007/s11306-013-0574-1
			Clinical and Metabolic Characterization of Lean Caucasian Subjects With Non-alcoholic Fatty Liver	Feldman, Alexandra	73	10.1038/ajg.2016.318
	64	insulin resistance	Quantitative Serum Nuclear Magnetic Resonance Metabolomics in Cardiovascular Epidemiology and	Soininen, Pasi	227	10.1161/CIRCGENETICS.114.000216
			Circulating branched-chain amino acid concentrations are associated with obesity and future insulin resistance in children and adolescents	McCormack, S E	209	10.1111/j.2047-6310.2012.00087.x
			A diabetes-predictive amino acid score and future cardiovascular disease	Magnusson, Martin	139	10.1093/eurheartj/ehs424
			Human metabolic correlates of body mass index	Moore, Steven C	87	10.1007/s11306-013-0574-1
			Clinical and Metabolic Characterization of Lean Caucasian Subjects With Non-alcoholic Fatty Liver	Feldman, Alexandra	73	10.1038/ajg.2016.318
			A diabetes-predictive amino acid score and future cardiovascular disease	Magnusson, Martin	139	10.1093/eurheartj/ehs424
			Human metabolic correlates of body mass index	Moore, Steven C	87	10.1007/s11306-013-0574-1

#1 obesity	60	risk	Metabolomics in Epidemiology: Sources of Variability in Metabolite Measurements and Implications	Sampson, Joshua N	83	10.1158/1055-9965.EPI-12-1109
			Metabolic biomarker signature to differentiate pancreatic ductal adenocarcinoma from chronic pancreatitis	Mayerle, Julia	53	10.1136/gutjnl-2016-312432
			Linking diet, physical activity, cardiorespiratory fitness and obesity to serum metabolite networks: findings from a population-based study	Floegel, A	52	10.1038/ijo.2014.39
	50	disease	Harmonizing lipidomics: NIST interlaboratory comparison exercise for lipidomics using SRM 1950-Metabolites in Frozen Human Plasma	Bowden, John A	120	10.1194/jlr.M079012
			Energy Metabolic Reprogramming in the Hypertrophied and Early Stage Failing Heart A Multisystems Approach	Lai, Ling	109	10.1161/CIRCHEARTFAILURE.114.001469
			Clinical and Metabolic Characterization of Lean Caucasian Subjects With Non-alcoholic Fatty Liver	Feldman, Alexandra	73	10.1038/ajg.2016.318
			Prognostic Implications of Long-Chain Acylcarnitines in Heart Failure and Reversibility With Mechanical Circulatory Support	Ahmad, Tariq	57	10.1016/j.jacc.2015.10.079
			Urinary metabolic profiles in early pregnancy are associated with preterm birth and fetal growth restriction in the Rhea mother-child cohort study	Maitre, Lea	45	10.1186/1741-7015-12-110
	43	amino acid	Quantitative Serum Nuclear Magnetic Resonance Metabolomics in Cardiovascular Epidemiology and	Soininen, Pasi	227	10.1161/CIRCGENETICS.114.000216
			A diabetes-predictive amino acid score and future cardiovascular disease	Magnusson, Martin	139	10.1093/eurheartj/ehs424
			Potential metabolite markers of schizophrenia	Yang, J	122	10.1038/mp.2011.131
			Long-term Leisure-time Physical Activity and Serum Metabolome	Kujala, Urho M	109	10.1161/CIRCULATIONAHA.112.105551

		Metabolomics as a Tool for Discovery of Biomarkers of Autism Spectrum Disorder in the Blood Plasma of Children	West, Paul R	93	10.1371/journal.pone.0112445
31	diet	Comparing metabolite profiles of habitual diet in serum and urine	Playdon, Mary C	59	10.3945/ajcn.116.135301
		Linking diet, physical activity, cardiorespiratory fitness and obesity to serum metabolite networks: findings from a population-based study	Floegel, A	52	10.1038/ijo.2014.39
		Influence of weight reduction on blood levels of C-reactive protein, tumor necrosis factor-alpha, interleukin-6, and oxylipins in obese subjects	Moeller, Katharina	24	10.1016/j.plefa.2015.12.001
		The impact of free or standardized lifestyle and urine sampling protocol on metabolome recognition accuracy	Wallner-Liebmann, Sandra	22	10.1007/s12263-014-0441-3
		Specific appetite, energetic and metabolomics responses to fat overfeeding in resistant-to-bodyweight-gain constitutional thinness	Germain, N	18	10.1038/nutd.2014.17
29	health	Combined Application of NMR- and GC-MS-Based Metabonomics Yields a Superior Urinary Biomarker Panel for Bipolar Disorder	Chen, Jian-jun	48	10.1038/srep05855
		Associations Between Metabolomic Compounds and Incident Heart Failure Among African Americans: The ARIC	Zheng, Yan	40	10.1093/aje/kwt004
		Novel Multimetabolite Prediction of Walnut Consumption by a Urinary Biomarker Model in a Free-Living Population: the PREDIMED Study	Garcia-Aloy, Mar	35	10.1021/pr500425r
		Effects of a Flavonoid-Rich Juice on Inflammation, Oxidative Stress, and Immunity in Elite Swimmers: A Metabolomics-Based Approach	Knab, Amy M	32	10.1123/ijsnem.23.2.150
		Objectively measured physical activity and plasma metabolomics in the Shanghai Physical Activity Study	Xiao, Qian	28	10.1093/ije/dyw033

	28	inflammation	Sex hormone-binding globulin associations with circulating lipids and metabolites and the risk for type 2 diabetes: observational and causal effect estimates	Wang, Qin	51	10.1093/ije/dyv093
			Breath metabolomic profiling by nuclear magnetic resonance spectroscopy in asthma	Ibrahim, B	41	10.1111/all.12211
			Purine metabolism is dysregulated in patients with major depressive disorder	Ali-Sisto, Toni	38	10.1016/j.psyneuen.2016.04.017
			Metabolomics-Based Analysis of Banana and Pear Ingestion on Exercise Performance and Recovery	Nieman, David C	31	10.1021/acs.jproteome.5b00909
			Molecular alterations in skeletal muscle in rheumatoid arthritis are related to disease activity, physical inactivity, and disability	Huffman, Kim M	28	10.1186/s13075-016-1215-7
	27	association	Meta-analysis of fecal metagenomes reveals global microbial signatures that are specific for colorectal cancer	Wirbel, Jakob	93	10.1038/s41591-019-0406-6
			Circulating Branched-Chain Amino Acids and Incident Cardiovascular Disease in a Prospective Cohort of US Women	Tobias, Deirdre K	33	10.1161/CIRCGEN.118.002157
			The ColoCare Study: A Paradigm of Transdisciplinary Science in Colorectal Cancer Outcomes	Ulrich, Cornelia M	15	10.1158/1055-9965.EPI-18-0773
			Phthalate exposure and childhood overweight and obesity: Urinary metabolomic evidence	Xia, Bin	14	10.1016/j.envint.2018.09.001
			LC-MS-based serum fingerprinting reveals significant dysregulation of phospholipids in chronic heart failure	Marcinkiewicz-Siemion, M	5	10.1016/j.jpba.2018.03.027
			Metabolomics as a Tool for Discovery of Biomarkers of Autism Spectrum Disorder in the Blood Plasma of Children	West, Paul R	93	10.1371/journal.pone.0112445

	41	performance	Influence of a Polyphenol-Enriched Protein Powder on Exercise-Induced Inflammation and Oxidative Stress in Athletes: A Randomized Trial Using a Metabolomics Approach	Nieman, David C	54	10.1371/journal.pone.0072215
			Metabolic biomarker signature to differentiate pancreatic ductal adenocarcinoma from chronic pancreatitis	Mayerle, Julia	53	10.1136/gutjnl-2016-312432
			Study of Induction Chemotherapy Efficacy in Oral Squamous Cell Carcinoma Using Pseudotargeted Metabolomics	Ye, Guozhu	53	10.1021/pr4011298
			The Muscle Metabolome Differs between Healthy and Frail Older Adults	Fazelzadeh, Parastoo	33	10.1021/acs.jproteome.5b00840
	33	oxidative stress	Metabolomics approach to assessing plasma 13-and 9-hydroxy-octadecadienoic acid and linoleic acid metabolite responses to 75-km cycling	Nieman, David C	38	10.1152/ajpregu.00092.2014
			Physical fitness level is reflected by alterations in the human plasma metabolome	Chorell, Elin	35	10.1039/c2mb05428k
			Bananas as an Energy Source during Exercise: A Metabolomics Approach	Nieman, David C	30	10.1371/journal.pone.0037479
			Targeted Metabolomics Connects Thioredoxin-interacting Protein (TXNIP) to Mitochondrial Fuel Selection and Regulation of Specific Oxidoreductase Enzymes in Skeletal Muscle	DeBalsi, Karen L	29	10.1074/jbc.M113.511535
			A pilot study comparing the metabolic profiles of elite-level athletes from different sporting disciplines	Al-Khelaifi, Fatima	27	10.1186/s40798-017-0114-z
			Prognostic Implications of Long-Chain Acylcarnitines in Heart Failure and Reversibility With Mechanical Circulatory Support	Ahmad, Tariq	57	10.1016/j.jacc.2015.10.079
			A pilot study comparing the metabolic profiles of elite-level athletes from different sporting disciplines	Al-Khelaifi, Fatima	27	10.1186/s40798-017-0114-z

	23	muscle	The Intersection of Aging Biology and the Pathobiology of Lung Diseases: A Joint NHLBI/NIA Workshop	Budinger, G R Scott	22	10.1093/gerona/glx090
			The ACTN3 R577X Polymorphism Is Associated with Cardiometabolic Fitness in Healthy Young Adults	Deschamps, Chelsea L	21	10.1371/journal.pone.0130644
			Understanding the response to endurance exercise using a systems biology approach: combining blood metabolomics, transcriptomics and miRNomics in horses	Mach, Nuria	17	10.1186/s12864-017-3571-3
	22	metabolome	The maternal-age-associated risk of congenital heart disease is modifiable	Schulkey, Claire E	39	10.1038/nature14361
			Effects of exposure to water disinfection by-products in a swimming pool: A metabolome-wide association study	van Veldhoven, Karin	27	10.1016/j.envint.2017.11.017
			Characterisation of the faecal metabolome and microbiome of Thoroughbred racehorses	Proudman, C J	25	10.1111/evj.12324
			Effects of sleep restriction on the human plasma metabolome	Bell, Lauren N	23	10.1016/j.physbeh.2013.08.007
			Predictive diagnosis of major depression using NMR-based metabolomics and least-squares support vector machine	Zheng, Hong	18	10.1016/j.cca.2016.11.039
	21	model	Dominant components of the Thoroughbred metabolome characterised by H-1-nuclear magnetic resonance spectroscopy: A metabolite atlas of common biofluids	Escalona, E E	21	10.1111/evj.12333
			Understanding the response to endurance exercise using a systems biology approach: combining blood metabolomics, transcriptomics and miRNomics in horses	Mach, Nuria	17	10.1186/s12864-017-3571-3
			PLS-Based and Regularization-Based Methods for the Selection of Relevant Variables in Non-targeted Metabolomics Data	Bujak, Renata	17	10.3389/fmolb.2016.00035 PG

#2 oxidative stress			Biomarkers in amyotrophic lateral sclerosis: combining metabolomic and clinical parameters to define disease	Blasco, H	12	10.1111/ene.12851
			Artificial Neural Network for Probabilistic Feature Recognition in Liquid Chromatography Coupled to High-Resolution Mass Spectrometry	Woldegebriel, Michael	12	10.1021/acs.analchem.6b03678
	21	response	Integrating Omic Technologies into Aquatic Ecological Risk Assessment and Environmental Monitoring: Hurdles, Achievements, and Future Outlook	Van Aggelen, Graham	99	10.1289/ehp.0900985
			H-1 NMR-Based Metabonomic Investigation of the Effect of Two Different Exercise Sessions on the Metabolic Fingerprint of Human Urine	Pechlivanis, Alexandros	80	10.1021/pr100684t
			H-1 NMR Study on the Short- and Long-Term Impact of Two Training Programs of Sprint Running on the Metabolic Fingerprint of Human Serum	Pechlivanis, Alexandros	47	10.1021/pr300846x
			Effects of a Flavonoid-Rich Juice on Inflammation, Oxidative Stress, and Immunity in Elite Swimmers: A Metabolomics-Based Approach	Knab, Amy M	32	10.1123/ijsnem.23.2.150
			Identification of serum analytes and metabolites associated with aerobic capacity	Lustgarten, Michael S	24	10.1007/s00421-012-2555-x
	15	physical exercise	Long-term Leisure-time Physical Activity and Serum Metabolome	Kujala, Urho M	109	10.1161/CIRCULATIONAHA.112.105551
			H-1 NMR-Based Metabonomic Investigation of the Effect of Two Different Exercise Sessions on the Metabolic Fingerprint of Human Urine	Pechlivanis, Alexandros	80	10.1021/pr100684t
			H-1 NMR-based metabolomics approach for exploring urinary metabolome modifications after acute and chronic physical exercise	Enea, C	64	10.1007/s00216-009-3289-4 PG 10

			H-1 NMR Study on the Short- and Long-Term Impact of Two Training Programs of Sprint Running on the Metabolic Fingerprint of Human Serum	Pechlivanis, Alexandros	47	10.1021/pr300846x
			Metabolomics investigation of exercise-modulated changes in metabolism in rat liver after exhaustive and endurance exercises	Huang, Chi-Chang	44	10.1007/s00421-009-1247-7
			Metabolomics Workbench: An international repository for metabolomics data and metadata, metabolite standards, protocols, tutorials and training, and analysis tools	Sud, Manish	175	10.1093/nar/gkv1042
			International NMR-Based Environmental Metabolomics Intercomparison Exercise	Viant, Mark R	112	10.1021/es802198z
	14	systems biology	Nuclear magnetic resonance-based metabolomics predicts exercise-induced ischemia in patients with suspected coronary artery disease	Barba, Ignasi	56	10.1002/mrm.21632
			Evaluation of regression models in metabolic physiology: predicting fluxes from isotopic data without knowledge of the pathway	Antoniewicz, Maciek R	33	10.1007/s11306-006-0018-2
			Whole Blood Transcriptomics and Urinary Metabolomics to Define Adaptive Biochemical Pathways of High-Intensity Exercise in 50-60 Year Old Masters Athletes	Mukherjee, Kamalika	25	10.1371/journal.pone.0092031
			Identification and Validation of Urinary Metabolite Biomarkers for Major Depressive Disorder	Zheng, Peng	126	10.1074/mcp.M112.021816
			A pilot study comparing the metabolic profiles of elite-level athletes from different sporting disciplines	Al-Khelaifi, Fatima	27	10.1186/s40798-017-0114-z
	13	stress	Identification of serum analytes and metabolites associated with aerobic capacity	Lustgarten, Michael S	24	10.1007/s00421-012-2555-x

			Metabolic time-course response after resistance exercise: A metabolomics approach	Berton, Ricardo	22	10.1080/02640414.2016.1218035
			Understanding the response to endurance exercise using a systems biology approach: combining blood metabolomics, transcriptomics and miRNomics in horses	Mach, Nuria	17	10.1186/s12864-017-3571-3
	9	liver	Effects of Intra- and Post-Operative Ischemia on the Metabolic Profile of Clinical Liver Tissue Specimens Monitored by NMR	Cacciatore, Stefano	22	10.1021/pr400702d
			Serum Metabolomics Analysis Reveals a Distinct Metabolic Profile of Patients with Primary Biliary Cholangitis	Hao, Juan	13	10.1038/s41598-017-00944-9
			Exercise-Induced Alterations in Skeletal Muscle, Heart, Liver, and Serum Metabolome Identified by Non-Targeted Metabolomics Analysis	Starnes, Joseph W	11	10.3390/metabo7030040
			AICAR stimulation metabolome widely mimics electrical contraction in isolated rat epitrochlearis muscle	Miyamoto, Licht	10	10.1152/ajpcell.00162.2013
			Serum metabolomics reveals the mechanistic role of functional foods and exercise for obesity management in rats	Ammar, Naglaa M	6	10.1016/j.jpba.2017.05.001
	21	protein	Serum metabolomics reveals gamma-glutamyl dipeptides as biomarkers for discrimination among different forms of liver disease	Soga, Tomoyoshi	145	10.1016/j.jhep.2011.01.031
			Comparative metabolomics of estrogen receptor positive and estrogen receptor negative breast cancer: alterations in glutamine and beta-alanine metabolism	Budczies, Jan	81	10.1016/j.jprot.2013.10.002
			Linking diet, physical activity, cardiorespiratory fitness and obesity to serum metabolite networks: findings from a population-based study	Floegel, A	52	10.1038/ijo.2014.39

			Metabolomic profiles as reliable biomarkers of dietary composition	Esko, Tonu	39	10.3945/ajcn.116.144428
			Diagnosis of major depressive disorder based on changes in multiple plasma neurotransmitters: a targeted metabolomics study	Pan, Jun-Xi	31	10.1038/s41398-018-0183-x
	13	supplementation	Metabolomics-Based Analysis of Banana and Pear Ingestion on Exercise Performance and Recovery	Nieman, David C	31	10.1021/acs.jproteome.5b00909
			Personalized Metabolomics for Predicting Glucose Tolerance Changes in Sedentary Women After High-Intensity Interval Training	Kuehnbaum, Naomi L	27	10.1038/srep06166
			Sportomics: Building a new concept in metabolic studies and exercise science	Bassini, Adriana	22	10.1016/j.bbrc.2013.12.137
			Metabolomics studies on db/db diabetic mice in skeletal muscle reveal effective clearance of overloaded intermediates by exercise	Xiang, Li	14	10.1016/j.aca.2017.11.082
			Metabolomics analysis of serum reveals the effect of Danggui Buxue Tang on fatigued mice induced by exhausting physical exercise	Miao, Xiaoyao	12	10.1016/j.jpba.2018.01.028
	11	nmr spectroscopy	Metabolomics provide new insights on lung cancer staging and discrimination from chronic obstructive pulmonary disease	Deja, Stanislaw	59	10.1016/j.jpba.2014.08.020
			Metabolomics study of esophageal adenocarcinoma	Zhang, Jian	47	0.1016/j.jtcvs.2010.08.025
			Metabolomics-Derived Prostate Cancer Biomarkers: Fact or Fiction?	Kumar, Deepak	37	10.1021/pr5011108
			Monitoring the Response of the Human Urinary Metabolome to Brief Maximal Exercise by a Combination of RP-UPLC-MS and H-1 NMR Spectroscopy	Pechlivanis, Alexandros	24	10.1021/acs.jproteome.5b00470

			Dominant components of the Thoroughbred metabolome characterised by H-1-nuclear magnetic resonance spectroscopy: A metabolite atlas of common biofluids	Escalona, E E	21	10.1111/evj.12333
			Metabolic Signatures of Adiposity in Young Adults: Mendelian Randomization Analysis and Effects of Weight Change	Wurtz, Peter	135	10.1371/journal.pmed.1001765
			Metabolomic Profiling of Serum from Human Pancreatic Cancer Patients Using H-1 NMR Spectroscopy and Principal Component Analysis	OuYang, Dong	68	10.1007/s12010-011-9240-0
	11	mortality	A family based tailored counselling to increase non-exercise physical activity in adults with a sedentary job and physical activity in their young children: design and methods of a year-long randomized controlled trial	Finni, Taija	19	10.1186/1471-2458-11-944
			Serum Metabolites Related to Cardiorespiratory Fitness, Physical Activity Energy Expenditure, Sedentary Time and Vigorous Activity	Wientzek, Angelika	10	10.1123/ijsnem.2013-0048
			A plasma metabolite panel as biomarkers for early primary breast cancer detection	Yuan, Baowen	9	10.1002/ijc.31996
			Linking diet, physical activity, cardiorespiratory fitness and obesity to serum metabolite networks: findings from a population-based study	Floegel, A	52	10.1038/ijo.2014.39
			Personalized nutrition and obesity	Qi, Lu	16	10.3109/07853890.2014.891802
	10	nutrition	Near-infrared spectroscopy and partial least squares-class modeling (PLS-CM) for metabolomics fingerprinting discrimination of intervention breakfasts ingested by obese individuals	Alvarez-Sanchez, Beatriz	8	10.1002/cem.2526
#3			Metabolomics profiling of xenobiotics in elite athletes: relevance to supplement consumption	Al-Khelaifi, Fatima	7	10.1186/s12970-018-0254-7

#3 hypertens-ion			Micronutrient Research, Programs, and Policy: From Meta-analyses to Metabolomics	Allen, Lindsay H	6	10.3945/an.113.005421
	10	m	Large-Scale Prediction of Collision Cross-Section Values for Metabolites in Ion Mobility-Mass Spectrometry	Zhou, Zhiwei	70	10.1021/acs.analchem.6b03091
			Metabolomics Data Normalization with EigenMS	Karpievitch, Yuliya V	43	10.1371/journal.pone.0116221
			Sample preparation and orthogonal chromatography for broad polarity range plasma metabolomics: Application to human subjects with neurodegenerative dementia	Armirotti, Andrea	24	10.1016/j.ab.2014.03.019
			Training in metabolomics research. II. Processing and statistical analysis of metabolomics data, metabolite identification, pathway analysis, applications of metabolomics and its future	Barnes, Stephen	21	0.1002/jms.3780
			Analysis of serum phospholipid profiles by liquid chromatography-tandem mass spectrometry in high resolution mode for evaluation of atherosclerotic patients	Calderon-Santiago, M	15	10.1016/j.chroma.2014.10.052
	8	pathway	Metabolomics Workbench: An international repository for metabolomics data and metadata, metabolite standards, protocols, tutorials and training, and analysis tools	Sud, Manish	175	10.1093/nar/gkv1042
			Diagnosis of major depressive disorder based on changes in multiple plasma neurotransmitters: a targeted metabolomics study	Pan, Jun-Xi	31	10.1038/s41398-018-0183-x
			Response of Gut Microbiota to Metabolite Changes Induced by Endurance Exercise	Zhao, Xia	26	10.3389/fmicb.2018.00765

			Training in metabolomics research. II. Processing and statistical analysis of metabolomics data, metabolite identification, pathway analysis, applications of metabolomics and its future	Barnes, Stephen	21	10.1002/jms.3780
			Metabolomics analysis of serum reveals the effect of Danggui Buxue Tang on fatigued mice induced by exhausting physical exercise	Miao, Xiaoyao	12	10.1016/j.jpba.2018.01.028
	8	survival	The ColoCare Study: A Paradigm of Transdisciplinary Science in Colorectal Cancer Outcomes	Ulrich, Cornelia M	15	10.1158/1055-9965.EPI-18-0773
			Associations of branched-chain amino acids with parameters of energy balance and survival in colorectal cancer patients: results from the ColoCare study	Delphan, Mahmoud	9	10.1007/s11306-017-1314-8
			Identification of metabolites in plasma for predicting survival in glioblastoma	Shen, Jie	6	10.1002/mc.22815
			LC-MS-based serum fingerprinting reveals significant dysregulation of phospholipids in chronic heart failure	Marcinkiewicz-Siemion, M	5	10.1016/j.jpba.2018.03.027
			A distinct glycerophospholipid metabolism signature of acute graft versus host disease with predictive value	Liu, Yue	3	10.1172/jci.insight.129494
	7	body mass index	Metabolic Signatures of Adiposity in Young Adults: Mendelian Randomization Analysis and Effects of Weight Change	Wurtz, Peter	135	10.1371/journal.pmed.1001765
			Associations of Body Mass and Fat Indexes With Cardiometabolic Traits	Bell, Joshua A	19	10.1016/j.jacc.2018.09.066
			Day-3 embryo metabolomics in the spent culture media is altered in obese women undergoing in vitro fertilization	Bellver, Jose	18	10.1016/j.fertnstert.2015.03.015
			Personalized nutrition and obesity	Qi, Lu	16	10.3109/07853890.2014.891802
			Associations of branched-chain amino acids with parameters of energy balance and survival in colorectal cancer patients: results from the ColoCare study	Delphan, Mahmoud	9	10.1007/s11306-017-1314-8

	5	proteomics	Sportomics: Building a new concept in metabolic studies and exercise science	Bassini, Adriana	22	10.1016/j.bbrc.2013.12.137
			MASSyPup - an 'Out of the Box' solution for the analysis of mass spectrometry data	Winkler, Robert	15	10.1002/jms.3314
			Rational and design of an overfeeding protocol in constitutional thinness: Understanding the physiology, metabolism and genetic background of resistance to weight gain	Ling, Yiin	8	10.1016/j.ando.2016.06.001
			Bridging the Gaps: The Promise of Omics Studies in Pediatric Exercise Research	Radom-Aizik, Shlomit	4	10.1123/pes.2015-0270
			Childhood obesity: a systems medicine approach	Stone, William L	2	10.2741/4441
	197	exercise	Investigations of the effects of gender, diurnal variation, and age in human urinary metabolomic profiles	Slupsky, Carolyn M	283	10.1021/ac0708588
			Circulating branched-chain amino acid concentrations are associated with obesity and future insulin resistance in children and adolescents	McCormack, S E	209	10.1111/j.2047-6310.2012.00087.x
			Long-term Leisure-time Physical Activity and Serum Metabolome	Kujala, Urho M	109	10.1161/CIRCULATIONAHA.112.105551
			Metabolomic profiling of dietary-induced insulin resistance in the high fat-fed C57BL/6J mouse	Shearer, J	94	10.1111/j.1463-1326.2007.00837.x
			A multivariate screening strategy for investigating metabolic effects of strenuous physical exercise in human serum	Pohjanen, Elin	85	10.1021/pr070007g
			Quantitative Serum Nuclear Magnetic Resonance Metabolomics in Cardiovascular Epidemiology and	Soininen, Pasi	227	10.1161/CIRCGENETICS.114.000216
			Targeted metabolomics for discrimination of systemic inflammatory disorders in critically ill patients	Schmerler, Diana	78	10.1194/jlr.P023309

	86	metabolism	Mass Spectrometry-Based Quantitative Metabolomics Revealed a Distinct Lipid Profile in Breast Cancer Patients	Qiu, Yunping	57	10.3390/ijms14048047
			Prognostic Implications of Long-Chain Acylcarnitines in Heart Failure and Reversibility With Mechanical Circulatory Support	Ahmad, Tariq	57	10.1016/j.jacc.2015.10.079
			Metabolomic Profiling Identifies Novel Circulating Biomarkers of Mitochondrial Dysfunction Differentially Elevated in Heart Failure With Preserved Versus Reduced Ejection Fraction: Evidence for Shared Metabolic Impairments in Clinical Heart Failure	Hunter, Wynn G	52	10.1161/JAHA.115.003190
	86	skeletal muscle	Energy Metabolic Reprogramming in the Hypertrophied and Early Stage Failing Heart A Multisystems Approach	Lai, Ling	109	10.1161/CIRCHEARTFAILURE.114.001469
			Medium Chain Acylcarnitines Dominate the Metabolite Pattern in Humans under Moderate Intensity Exercise and Support Lipid Oxidation	Lehmann, Rainer	81	10.1371/journal.pone.0011519
			Muscle-specific loss of Bmal1 leads to disrupted tissue glucose metabolism and systemic glucose homeostasis	Harfmann, Brianna D	70	10.1186/s13395-016-0082-x
			Metabolite signatures of exercise training in human skeletal muscle relate to mitochondrial remodelling and cardiometabolic fitness	Huffman, Kim M	53	10.1007/s00125-014-3343-4
			Six weeks of a polarized training-intensity distribution leads to greater physiological and performance adaptations than a threshold model in trained cyclists	Neal, Craig M	52	10.1152/japplphysiol.00652.2012
			Metabolic and hormonal responses to isoenergetic high-intensity interval exercise and continuous moderate-intensity exercise	Peake, Jonathan M	70	10.1152/ajpendo.00276.2014

#4 skeletal muscle	18	glucose	Impact of combined resistance and aerobic exercise training on branched-chain amino acid turnover, glycine metabolism and insulin sensitivity in overweight humans	Glynn, Erin L	50	10.1007/s00125-015-3705-6
			Serum metabolic profiles in overweight and obese women with and without metabolic syndrome	Wiklund, Petri K	42	10.1186/1758-5996-6-40
			Metabolomics Approach for Analyzing the Effects of Exercise in Subjects with Type 1 Diabetes Mellitus	Brugnara, Laura	41	10.1371/journal.pone.0040600
			A Protein-Leucine Supplement Increases Branched-Chain Amino Acid and Nitrogen Turnover But Not Performance	Nelson, Andre R	31	10.1249/MSS.0b013e3182290371
	15	validation	Identification of novel candidate plasma metabolite biomarkers for distinguishing serous ovarian carcinoma and benign serous ovarian tumors	Buas, Matthew F	29	10.1016/j.ygyno.2015.10.021
			The Cooperative Health Research in South Tyrol (CHRIS) study: rationale, objectives, and preliminary results	Pattaro, Cristian	22	10.1186/s12967-015-0704-9
			Metabolite signature for diagnosing major depressive disorder in peripheral blood mononuclear cells	Zheng, Peng	19	10.1016/j.jad.2016.02.008
			Global profiling of the muscle metabolome: method optimization, validation and application to determine exercise-induced metabolic effects	Alves, Rodrigo D A M	10	10.1007/s11306-014-0701-7
			Metabolic profiling identifies phospholipids as potential serum biomarkers for schizophrenia	Wang, Dongfang	10	10.1016/j.psychres.2018.12.008
			Energy Metabolic Reprogramming in the Hypertrophied and Early Stage Failing Heart A Multisystems Approach	Lai, Ling	109	10.1161/CIRCHEARTFAILURE.114.001469

	14	gene expression	Predictive Metabolomics Evaluation of Nutrition-Modulated Metabolic Stress Responses in Human Blood Serum During the Early Recovery Phase of Strenuous Physical Exercise	Chorell, Ellin	78	10.1021/pr900081q
			Molecular alterations in skeletal muscle in rheumatoid arthritis are related to disease activity, physical inactivity, and disability	Huffman, Kim M	28	10.1186/s13075-016-1215-7
			GC-MS based metabolomics identification of possible novel biomarkers for schizophrenia in peripheral blood mononuclear cells	Liu, Mei-Ling	28	10.1039/c4mb00157e
			Whole Blood Transcriptomics and Urinary Metabolomics to Define Adaptive Biochemical Pathways of High-Intensity Exercise in 50-60 Year Old Masters Athletes	Mukherjee, Kamalika	25	10.1371/journal.pone.0092031
	11	database	Large-Scale Prediction of Collision Cross-Section Values for Metabolites in Ion Mobility-Mass Spectrometry	Zhou, Zhiwei	70	10.1021/acs.analchem.6b03091
			Fast metabolite identification with Input Output Kernel Regression	Brouard, Celine	28	10.1093/bioinformatics/btw246
			Whole-body fat oxidation increases more by prior exercise than overnight fasting in elite endurance athletes	Hall, Ulrika Andersson	15	10.1139/apnm-2015-0452
			Development and Validation of a High-Throughput Mass Spectrometry Based Urine Metabolomic Test for the Detection of Colonic Adenomatous Polyps	Deng, Lu	13	10.3390/metabo7030032
			Liquid Chromatography-Mass Spectrometry-Based Metabolomics of Nonhuman Primates after 4 Gy Total Body Radiation Exposure: Global Effects and Targeted Panels	Pannkuk, Evan L	9	10.1021/acs.jproteome.9b00101
			Six weeks of a polarized training-intensity distribution leads to greater physiological and performance adaptations than a threshold model in trained cyclists	Neal, Craig M	52	10.1152/japplphysiol.00652.2012

	9	capacity	Metabolomics Approach for Analyzing the Effects of Exercise in Subjects with Type 1 Diabetes Mellitus	Brugnara, Laura	41	10.1371/journal.pone.0040600
			Effects of a Flavonoid-Rich Juice on Inflammation, Oxidative Stress, and Immunity in Elite Swimmers: A Metabolomics-Based Approach	Knab, Amy M	32	10.1123/ijsnem.23.2.150
			Plasma metabolic profile in COPD patients: effects of exercise and	Rodriguez, Diego A	21	10.1007/s11306-011-0336-x
			Reduced mitochondrial lipid oxidation leads to fat accumulation in myosteatosis	Gumucio, Jonathan P	15	10.1096/fj.201802457RR
	7	oxidation	Six weeks of a polarized training-intensity distribution leads to greater physiological and performance adaptations than a threshold model in trained cyclists	Neal, Craig M	52	10.1152/japplphysiol.00652.2012
			Type 2 diabetes alters metabolic and transcriptional signatures of glucose and amino acid metabolism during exercise and recovery	Hansen, Jakob S	43	10.1007/s00125-015-3584-x
			The relationship between aerobic fitness level and metabolic profiles in healthy adults	Morris, Ciara	35	10.1002/mnfr.201200629
			Metabolic modeling of muscle metabolism identifies key reactions linked to insulin resistance phenotypes	Nogiec, Christophe r	18	10.1016/j.molmet.2014.12.012
			Metabolomics studies on db/db diabetic mice in skeletal muscle reveal effective clearance of overloaded intermediates by exercise	Xiang, Li	14	10.1016/j.aca.2017.11.082
	6	gene	Untargeted 1H-NMR metabolomics in CSF	Blasco, Helene	29	10.1212/WNL.0000000000000274
			Estrogen-Related Receptor-alpha Coordinates Transcriptional Programs Essential for Exercise Tolerance and Muscle Fitness	Perry, Marie-Claude	21	10.1210/me.2014-1281
			Metabolomics biomarkers to predict acamprosate treatment response in alcohol-dependent subjects	Hinton, David J	11	10.1038/s41598-017-02442-4

			A mutation in Ampd2 is associated with nephrotic syndrome and hypercholesterolemia in mice	Helmering, Joan	10	10.1186/1476-511X-13-167
			MetaComp: comprehensive analysis software for comparative meta-omics including comparative metagenomics	Zhai, Peng	8	10.1186/s12859-017-1849-8
	56	metabolite	Metabolic and hormonal responses to isoenergetic high-intensity interval exercise and continuous moderate-intensity exercise	Peake, Jonathan M	70	10.1152/ajpendo.00276.2014
			Identifying biomarkers of dietary patterns by using metabolomics	Playdon, Mary C	62	10.3945/ajcn.116.144501
			Comparing metabolite profiles of habitual diet in serum and urine	Playdon, Mary C	59	10.3945/ajcn.116.135301
			Influence of a Polyphenol-Enriched Protein Powder on Exercise-Induced Inflammation and Oxidative Stress in Athletes: A Randomized Trial Using a Metabolomics Approach	Nieman, David C	54	10.1371/journal.pone.0072215
			Metabolomics Study of Urine in Autism Spectrum Disorders Using a Multiplatform Analytical Methodology	Dieme, Binta	50	10.1021/acs.jproteome.5b00699
	18	rat	Monitoring the Response of the Human Urinary Metabolome to Brief Maximal Exercise by a Combination of RP-UPLC-MS and H-1 NMR Spectroscopy	Pechlivanis, Alexandros	24	10.1021/acs.jproteome.5b00470
			Effects of Acute Systematic Hypoxia on Human Urinary Metabolites Using LC-MS-Based Metabolomics	Lou, Bih-Show	19	10.1089/ham.2013.1130
			Beneficial effects of exercise on gut microbiota functionality and barrier integrity, and gut-liver crosstalk in an in vivo model of early obesity and non-alcoholic fatty liver disease	Carbajo-Pescador, Sara	16	10.1242/dmm.039206
			Molecular and metabolomic effects of voluntary running wheel activity on skeletal muscle in late middle-aged rats	Garvey, Sean M	15	10.14814/phy2.12319

			Metabolomics analysis of serum reveals the effect of Danggui Buxue Tang on fatigued mice induced by exhausting physical exercise	Miao, Xiaoyao	12	10.1016/j.jpba.2018.01.028
	17	blood	Metabolic time-course response after resistance exercise: A metabolomics approach	Berton, Ricardo	22	10.1080/02640414.2016.1218035
			Individual Human Metabolic Phenotype Analyzed by H-1 NMR of Saliva Samples	Wallner-Liebniann, Sandra	18	10.1021/acs.jproteome.5b01060
			Human Genetic Variation, Sport and Exercise Medicine, and Achilles Tendinopathy: Role for Angiogenesis-Associated Genes	Rahim, Masouda	14	10.1089/omi.2016.0116
			Targeted Metabolomics Approach To Detect the Misuse of Steroidal Aromatase Inhibitors in Equine Sports by Biomarker Profiling	Chan, George Ho Man	11	10.1021/acs.analchem.5b03165
			A plasma metabolite panel as biomarkers for early primary breast cancer detection	Yuan, Baowen	9	10.1002/ijc.31996
	17	prediction	Metabolomics insights into early type 2 diabetes pathogenesis and detection in individuals with normal fasting glucose	Merino, Jordi	31	10.1007/s00125-018-4599-x
			Fast metabolite identification with Input Output Kernel Regression	Brouard, Celine	28	10.1093/bioinformatics/btw246
			Maternal urinary metabolic signatures of fetal growth and associated clinical and environmental factors in the INMA study	Maitre, Lea	25	10.1186/s12916-016-0706-3
			Efficiency of genomic selection for tomato fruit quality	Duangjit, Janejira	23	10.1007/s11032-016-0453-3
			A Pharmacometabonomic Approach To Predicting Metabolic Phenotypes and Pharmacokinetic Parameters of Atorvastatin in Healthy Volunteers	Huang, Qing	21	10.1021/acs.jproteome.5b00440
			Investigation of Gender-Specific Exhaled Breath Volatome in Humans by GCxGC-TOF-MS	Das, Mrinal Kumar	39	10.1021/ac403541a

#5 aging	16	age	NMR-Based Metabolomic Profiling of Overweight Adolescents: An Elucidation of the Effects of Inter-/Intraindividual Differences, Gender, and Pubertal Development	Zheng, Hong	23	10.1155/2014/537157
			The Intersection of Aging Biology and the Pathobiology of Lung Diseases: A Joint NHLBI/NIA Workshop	Budinger, G R Scott	22	10.1093/gerona/glx090
			Phthalate exposure and childhood overweight and obesity: Urinary metabolomic evidence	Xia, Bin	14	10.1016/j.envint.2018.09.001
			Characterization of trotter horses urine metabolome by means of proton nuclear magnetic resonance spectroscopy	Zhu, Chenglin	11	10.1007/s11306-018-1403-3
	13	gut microbiota	Changes in intestinal microbiota composition and metabolism coincide with increased intestinal permeability in young adults under prolonged physiological stress	Karl, J Philip	77	10.1152/ajpgi.00066.2017
			Response of Gut Microbiota to Metabolite Changes Induced by Endurance Exercise	Zhao, Xia	26	10.3389/fmicb.2018.00765
			Urinary biomarker panel for diagnosing patients with depression and anxiety disorders	Chen, Jian-jun	23	10.1038/s41398-018-0245-0
			Effect of aerobic exercise and low carbohydrate diet on pre-diabetic non-alcoholic fatty liver disease in postmenopausal women and middle aged men - the role of gut microbiota composition: study protocol for the AELC randomized controlled trial	Liu, Wu Yi	19	10.1186/1471-2458-14-48
			Intestinal Metagenomes and Metabolomes in Healthy Young Males: Inactivity and Hypoxia Generated Negative Physiological Symptoms Precede Microbial Dysbiosis	Sket, Robert	11	10.3389/fphys.2018.00198

12	aging	The effects of graded levels of calorie restriction: IX. Global metabolomic screen reveals modulation of carnitines, sphingolipids and bile acids in the liver of C57BL/6 mice	Green, Cara L	21	10.1111/acel.12570
		Improved motor and cognitive performance with sodium nitrite supplementation is related to small metabolite signatures: a pilot trial in middle-aged and older adults	Justice, Jamie N	19	10.18632/aging.100842
		Molecular and metabolomic effects of voluntary running wheel activity on skeletal muscle in late middle-aged rats	Garvey, Sean M	15	10.14814/phy2.12319
		Impact of exercise on fecal and cecal metabolome over aging: a longitudinal study in rats	Deda, Olga	11	10.4155/bio-2016-0222
		Impact of Exercise and Aging on Rat Urine and Blood Metabolome. An LC-MS Based Metabolomics Longitudinal Study	Deda, Olga	7	10.3390/metabo7010010
8	carnitine	Metabolomics studies on db/db diabetic mice in skeletal muscle reveal effective clearance of overloaded intermediates by exercise	Xiang, Li	14	10.1016/j.aca.2017.11.082
		Circulating Metabolites Associated with Alcohol Intake in the European Prospective Investigation into Cancer and Nutrition Cohort	van Roekel, Eline H	6	10.3390/nu10050654
		LC-MS-based serum fingerprinting reveals significant dysregulation of phospholipids in chronic heart failure	Marcinkiewicz-Siemion, M	5	10.1016/j.jpba.2018.03.027
		Metabolomic Response to Acute Hypoxic Exercise and Recovery in Adult Males	Davison, Gareth	4	10.3389/fphys.2018.01682
		An NMR-Based Approach to Identify Urinary Metabolites Associated with Acute Physical Exercise and Cardiorespiratory Fitness in Healthy Humans-Results of the KarMeN Study	Kistner, Sina	0	10.3390/metabo10050212

	7	lipidomics	Modulation of the lipidomic profile due to a lipid challenge and fitness level: a postprandial study	Morris, Ciara	7	10.1186/s12944-015-0062-x
			Comparative determination of fatty acid composition of low-molecular components of blood plasma by three mass spectrometry techniques: the 'old-new' exercise in lipidomics	Milman, B L	2	10.1134/S1061934815140099
			Physical activity and lipidomics in a population at high risk of type 2 diabetes mellitus	Henson, Joseph	1	10.1080/02640414.2020.1744836
			Plasma Metabolomics Profiles are Associated with the Amount and Source of Protein Intake: A Metabolomics Approach within the PREDIMED Study	Hernandez-Alonso, Pablo	0	10.1002/mnfr.202000178
			Comprehensive Metabolomic and Lipidomic Analysis Reveals Metabolic Changes After Mindfulness Training	Chen, Wei	0	10.1007/s12671-020-01359-w
	6	targeted metabolomics	Harmonizing lipidomics: NIST	Bowden,	120	10.1194/jlr.M079012
			Food Targeting: Geographical Origin Determination of Hazelnuts (Corylus avellana) by LC-QqQ-MS/MS-Based Targeted Metabolomics Application	Klockmann, Sven	24	10.1021/acs.jafc.6b05007
			Identification of metabolic pathway disturbances using multimodal metabolomics in autistic disorders in a Middle Eastern population	Bitar, Tania	18	10.1016/j.jpba.2018.01.007
			Impact of Exercise and Aging on Rat Urine and Blood Metabolome. An LC-MS Based Metabolomics Longitudinal Study	Deda, Olga	7	10.3390/metabo7010010
			Circulating Metabolites Associated with Alcohol Intake in the European Prospective Investigation into Cancer and Nutrition Cohort	van Roekel, Eline H	6	10.3390/nu10050654
