# Supplementary material for: Research in the Field of Exercise and Metabolomics: A Bibliometric and Visual Analysis
Source: Metabolites. 2022 Jun 14;12(6):542. doi: 10.3390/metabo12060542 (PMC9230385; doi:10.3390/metabo12060542)
Supplement: Supplementary file 1 [file metabolites-12-00542-s001.zip › Supplementary Table S2.pdf]

Supplementary Table S2. Top 20 authors distributed by publications in the field of exercise and metabolomics

Rank	First publication year	Author	publications	Institution
1	2013	PENG ZHENG	10	1. Department of Neurology, The First Affiliated Hospital of Chongqing Medical University.<br>2. Institute of Neuroscience and the Collaborative Innovation Center for Brain Science, Chongqing Medical University.<br>3. Chongqing Key Laboratory of Neurobiology.
2	2013	PENG XIE	10	1. Department of Neurology, The First Affiliated Hospital of Chongqing Medical University.<br>2. Institute of Neuroscience and the Collaborative Innovation Center for Brain Science, Chongqing Medical University.<br>3. Chongqing Key Laboratory of Neurobiology.
3	2015	HANS J VOGEL	8	1. North Carolina Research Campus, Appalachian State University.
4	2017	CLARY B CLISH	8	1. Broad Institute of MIT and Harvard University Cambridge.
5	2013	DAVID C NIEMAN	8	1. Human Performance Laboratory, Appalachian State University.
6	2014	OLIVER FIEHN	8	1. West Coast Metabolomics Center, University of California.<br>2. Biochemistry Department, King Abdulaziz University.
7	2019	LIMING LIANG	5	1. Department of Epidemiology, Harvard T.H. Chan School of Public Health.<br>2. Department of Biostatistics, Harvard T.H. Chan School of Public Health.
8	2016	JOSHUA N SAMPSON	5	1. Division of Cancer Epidemiology and Genetics, National Cancer Institute, NIH, Department of Health and Human Services.
9	2018	ROBERT E GERSZTEN	5	1. Beth Israel Deaconess Medical Center, Harvard Medical School.
10	2018	KARSTEN SUHRE	5	1. Weill Cornell Medicine- Qatar.
11	2013	R ANDREW SHANELY	5	1. Appalachian State University, Human Performance Lab, North Carolina Research Campus

12	2019	JOANN E MANSON	5	1.Division of Preventive Medicine, Department of Medicine, Brigham and Women's Hospital.<br>2.Harvard Medical School, Boston, MA, USA; 3Department of Biostatistics and Epidemiology, School of Public Health and Health Sciences, University of Massachusetts.<br>3.Department of Nutrition, Harvard TH Chan School of Public Health.<br>4.Department of Epidemiology, Harvard TH Chan School of Public Health.
13	2018	FRANCESCO DONATI	4	1.Laboratorio Antidoping, Federazione Medico Sportiva Italiana.
14	2014	RICHARD WILSON	4	1.Central Science Laboratory, University of Tasmania.
15	2016	STEVEN C MOORE	4	1.Nutritional Epidemiology Branch, Division of Cancer Epidemiology and Genetics, National Cancer Institute, National Institutes of Health.
16	2019	KATHRYN M REXRODE	4	1.Division of Preventive Medicine, Department of Medicine, Brigham and Women's Hospital.<br>2.Harvard Medical School, Boston, MA, USA; 3Department of Biostatistics and Epidemiology, School of Public Health and Health Sciences, University of Massachusetts.<br>3.Division of Women's Health, Department of Medicine, Brigham and Women's Hospital.
17	2018	COSTAS GEORGAKOPOULOS	4	1.Anti Doping Laboratory Qatar, Sports City.
18	2017	LAURENCE LE MOYEC	4	1. UBIAE EA 7362, Université Evry, Université Paris-Saclay.
19	2015	WILLIAM E KRAUS	4	1.Duke University.
20	2013	YING WANG	4	1.Department of Neurology, The First Affiliated Hospital of Chongqing Medical University.<br>2.Institute of Neuroscience and the Collaborative Innovation Center for Brain Science, Chongqing Medical University.<br>3. Chongqing Key Laboratory of Neurobiology.
