# Supplementary material for: Research in the Field of Exercise and Metabolomics: A Bibliometric and Visual Analysis
Source: Metabolites. 2022 Jun 14;12(6):542. doi: 10.3390/metabo12060542 (PMC9230385; doi:10.3390/metabo12060542)
Supplement: Supplementary file 1 [file metabolites-12-00542-s001.zip › Supplementary Table S3.pdf]

Supplementary Table S3. Top 20 authors distributed by citations in the field of exercise and metabolomics

Rank	First cited year	Cited author	Cited frequency	Institution
1	2007	Nicholson JK	115	1.Australian National Phenome Centre, Health Futures Institute, Murdoch University.<br>2.Center for Computational and Systems Medicine, Health Futures Institute, Murdoch University.
2	2009	Wishart DS	100	1.Department of Biological Sciences, University of Alberta.
3	2010	Lewis GD	81	1.Cardiology Division of Massachusetts General Hospital
4	2014	Xia JG	71	1.Institute of Parasitology, McGill University.<br>2.Department of Human Genetics, McGill University.<br>3.Department of Animal Science, McGill University.
5	2014	BENJAMINI Y	69	1.Sagol School of Neuroscience, Tel-Aviv University.<br>2.Department of Statistics and Operations Research, Tel-Aviv University.
6	2013	Wang TJ	64	1.Division of Cardiovascular Medicine, Vanderbilt University Medical Center.
7	2013	Newgard CB	62	1.Sarah W. Stedman Nutrition and Metabolism Center, Duke University Medical Center.<br>2.Duke Molecular Physiology Institute, Duke University Medical Center.<br>3.Department of Pharmacology & Cancer Biology, Duke University Medical Center.<br>4.Department of Medicine, Divisions of Endocrinology & Metabolism and Cardiology, Duke University Medical Center.
8	2005	Fiehn O	50	1.West Coast Metabolomics Center, University of California.

9	2006	Dunn WB	48	1.Manchester Centre for Integrative Systems Biology, University of Manchester.<br>2. Department of Chemistry, Manchester Interdisciplinary Biocentre, University of Manchester.<br>3.Centre for Advanced Discovery and Experimental Therapeutics,Manchester Biomedical Research Centre.
10	2012	Pechlivanis A	46	1.Department of Chemistry, Aristotle University of Thessalonik.
11	2014	Smith CA	43	1.Hall-Atwater Laboratories, Wesleyan University.<br>2.Department for Theoretical and Computational Biophysics, Max Planck Institute for Biophysical Chemistry.
12	2006	Trygg J	39	1.Sartorius Corporate Research.<br>2.Computational Life Science Cluster (CLiC), Umeå University.
13	2013	Nieman DC	38	1.Appalachian State University
14	2009	Chorell E	36	1.Department of Public Health and Clinical Medicine, Umeå University.
15	2013	Floegel A	36	1.Department of Epidemiology, German Institute of Human Nutrition Potsdam-[illegible]
16	2007	Holmes E	35	1.Australian National Phenome Centre, Health Futures Institute, Murdoch University.<br>2.Center for Computational and Systems Medicine, Health Futures Institute, Murdoch University.<br>3.Section for Nutrition Research, Imperial College London.
17	2009	Yan B	35	1.School of Environmental Science and Engineering, Tianjin University.<br>2.Key Laboratory of Biomass-based Oil and Gas (Tianjin University), China Petroleum and Chemical Industry Federation.
18	2013	Huffman KM	35	1.Duke Molecular Physiology Institute, Duke University,.<br>2.Department of Medicine, Duke University School of Medicine, Durham
19	2008	Lindon JC	34	1.Biological Chemistry, Biomedical Sciences Division, Faculty of Medicine, Imperial College of Science, Technology and Medicine

20	2016	SUMNER LW	33	1.Department of Biochemistry, University of Missouri.<br>2.Metabolomics and Bond Life Sciences Centers, University of Missouri.
